# Boosting Ferroelectricity: 2D and Polymer Ferroelectric Hybrids Enabling Ambipolar Nonvolatile MoS_2_ Memory Transistor

**DOI:** 10.1002/advs.76127

**Published:** 2026-06-15

**Authors:** Yeonsu Jeong, Honglei Wang, Pietro Tordi, Ying Chieh Hu, Adrián Tamayo, Bing Wu, Hyun Soo Ahn, Zdenek Sofer, Jong Hoon Jung, Massimo Bonini, Paolo Samorì

**Affiliations:** ^1^ Institute of Quantum Science Department of Physics Inha University Incheon South Korea; ^2^ University of Strasbourg & CNRS ISIS & icFRC Strasbourg France; ^3^ Department of Chemistry “Ugo Schiff” and CSGI University of Florence Florence Italy; ^4^ Department of Inorganic Chemistry University of Chemistry and Technology Prague Prague Czech Republic; ^5^ Program in Semiconductor and Device Inha University Incheon South Korea

**Keywords:** ambipolar memory, CuInP_2_S_6_, hybrid ferroelectrics, MoS_2_, P(VDF‐TrFE)

## Abstract

The rise of data‐driven technologies has exposed the limitations of von Neumann architectures in meeting the growing demands for efficient data storage and processing. These challenges motivate the development of ferroelectric materials that combine strong polarization, reliable switching, and multifunctionality. Here, we report a hybrid ferroelectric platform that integrates two‐dimensional (2D) CuInP_2_S_6_ (CIPS) nanosheets into a poly(vinylidene fluoride–trifluoroethylene) (P(VDF‐TrFE)) matrix, enabling cooperative ferroelectric–ferroelectric coupling. A solvent‐balanced isopropanol–methyl ethyl ketone process ensures uniform CIPS dispersion and promotes ferroelectric β‐phase crystallization of P(VDF‐TrFE), resulting in synergistic dipole reinforcement. Consequently, the hybrid film exhibits a nearly 75% increase in remnant polarization and a 15% reduction in coercive field. Leveraging this enhanced ferroelectricity, MoS_2_‐based ferroelectric transistors demonstrate stable nonvolatile memory characteristics, exhibiting reliable retention beyond 10,000 s and endurance over 300 switching cycles. Intriguingly, the hybrid dielectric further enables high‐performance unipolar p‐type operation under extended negative gating. Moreover, optoelectronic multilevel memory behavior emerges from the cooperative interplay between the dipole orientations in P(VDF‐TrFE) and photon‐induced partial depolarization of CIPS. This work represents the first demonstration of polarization‐cooperative enhancement in polymer ferroelectrics using 2D ferroelectric fillers and establishes P(VDF‐TrFE)/CIPS hybrids as a versatile platform for flexible, low‐power, and multifunctional ferroelectric electronics.

## Graphical abstract

Two‐dimensional CuInP_2_S_6_ nanosheets are incorporated into a P(VDF‐TrFE) matrix to induce polarization‐cooperative ferroelectric coupling. The resulting P(VDF‐TrFE)/CuInP_2_S_6_ hybrid film exhibits reinforced ferroelectric ordering and reduced coercive electric fields compared with pristine P(VDF‐TrFE). These improvements lead to superior nonvolatile operation, unipolar p‐type transport, and optoelectronically addressable multilevel memory in MoS_2_‐based ferroelectric memory transistors.

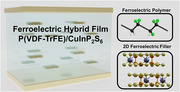

## 1 Introduction

The explosive growth of data‐driven technologies such as artificial intelligence and machine learning has triggered an unprecedented demand for high‐density, fast, and energy‐efficient nonvolatile memory devices [1, 2, 3, 4, 5, 6, 7]. Among various material platforms in the post‐Moore era [8, 9, 10], ferroelectric polymers have attracted extensive interest owing to their intrinsic switchable polarization, low‐temperature processability, mechanical flexibility, and solution‐based printability, offering a promising route toward downscaled, lightweight, and flexible memory systems for the emerging More‐than‐Moore technologies [11, 12, 13, 14, 15, 16, 17, 18, 19]. In particular, poly(vinylidene fluoride–trifluoroethylene) [P(VDF‐TrFE)] has been widely explored as a prototypical ferroelectric polymer due to its well‐defined ferroelectric phase and ease of integration with diverse substrates [17, 18, 19, 20, 21, 22, 23, 24].

However, the relatively low spontaneous polarization and dielectric constant of P(VDF‐TrFE), compared to high‐k ceramic ferroelectric dielectrics, have severely limited its application in high‐performance and reliable nonvolatile memories [25, 26, 27]. To overcome these intrinsic drawbacks, various conductive or insulating fillers have been incorporated into the polymer matrix to enhance dipole alignment or improve dielectric and mechanical properties. Conductive fillers such as carbon nanotubes, graphene, or MXenes tend to form percolation networks that lead to excessive leakage currents, reduced breakdown strength, and unstable polarization retention [28, 29, 30]. In addition, the metallic nature of these fillers can distort the local electric field distribution, suppressing uniform dipole switching in the polymer matrix. On the other hand, oxide fillers such as BaTiO_3_, PbTiO_3_, or HfOx possess high dielectric constants but suffer from poor interfacial compatibility with the fluoropolymer chains [31, 32, 33, 34]. The ionic bonding character and rigid lattice of these oxides induce charge trapping, interfacial defects, and significant mechanical mismatch, which degrade the flexibility and long‐term reliability of the multicomponent films. These limitations highlight the need for a fundamentally different strategy that can reinforce polymer ferroelectricity through intrinsic physical coupling, rather than relying on electrically or structurally incompatible fillers.

In this context, we propose a new design concept that integrates two‐dimensional (2D) ferroelectric fillers into ferroelectric polymer matrices to realize synergistic polarization coupling. Unlike conventional conductive or insulating fillers, 2D ferroelectrics can intrinsically interact with the host polymer via ferroelectric dipole–dipole coupling, promoting interfacial domain ordering without sacrificing electrical insulation or mechanical flexibility. Among various candidates, CuInP_2_S_6_ (CIPS) stands out as a layered van der Waals (vdW) ferroelectric material possessing robust out‐of‐plane polarization, high thermal stability, and atomically thin architecture ideally suited for integration with ferroelectric polymers [35, 36, 37, 38, 39].

Herein, we report a hybrid ferroelectric material combining P(VDF‐TrFE) and liquid phase exfoliated CIPS nanosheets, in which ferroelectric–ferroelectric coupling induces cooperative dipole reinforcement and substantial modulation of the coercive field (*E*_C_). The incorporation of ultrathin CIPS flakes promotes the formation of the ferroelectric β‐phase in P(VDF‐TrFE), enabling markedly enhanced dipole alignment. Polarization–electric field (*P*–*E*) measurements further confirm that the remnant polarization of the hybrid increases by nearly 75%, providing unambiguous evidence for the strong polarization reinforcement induced by the 2D ferroelectric fillers. Leveraging this enhanced ferroelectricity, we demonstrate a MoS_2_‐based ferroelectric memory transistor that exhibits bidirectional carrier transport, achieving electron and hole mobilities of up to 44.35 and 0.34 cm^2^ V^−1^ s^−1^, respectively. The device exhibits stable photodetection across multiple combinations of wavelength (680, 530, and 455 nm) and carrier accumulation states in MoS_2_ (electron and hole accumulation), showing a photoresponse as high as 26,400 A W^−1^ under 455 nm illumination in the electron accumulation state. Intriguingly, when 455 nm illumination is applied to the memory transistor in the hole‐accumulated state, it induces partial depolarization within the CIPS nanosheets, thereby enabling optoelectronic multilevel memory behavior. This unique polarization–photon interaction demonstrates the rich optoelectronic tunability unlocked by the hybrid ferroelectric architecture. Collectively, these results establish P(VDF‐TrFE)/CIPS hybrids as a multifunctional platform for high‐performance ferroelectric and optoelectronic memory devices. This study represents the first demonstration of polarization‐cooperative enhancement in polymer ferroelectrics via 2D ferroelectric fillers, offering a new paradigm for the design of next‐generation ferroelectric electronics.

## 2 Results and Discussion

**FIGURE 1:**
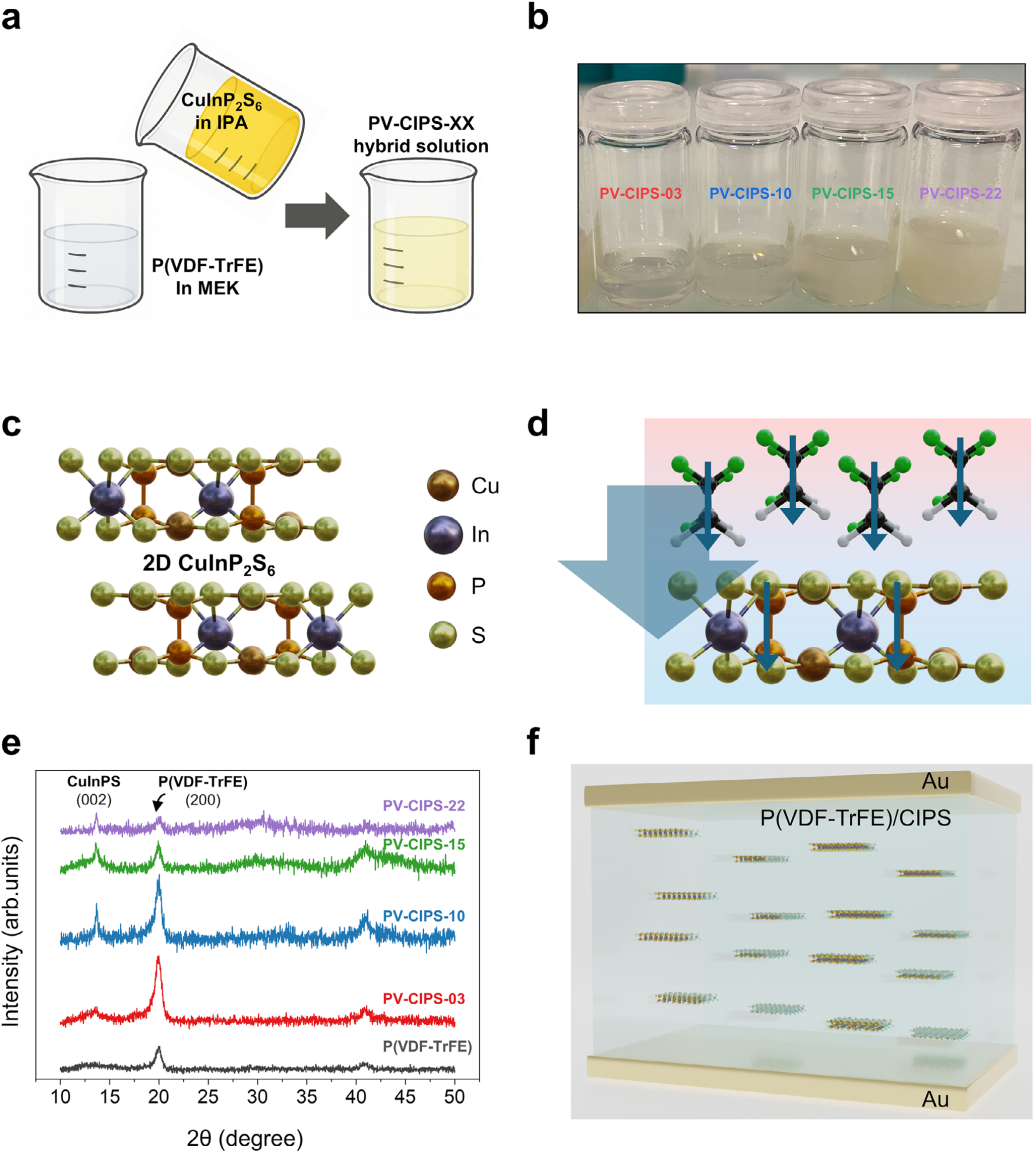
Preparation and structural characterization of P(VDF‐TrFE)/CIPS hybrids. (a) Illustration of the hybrid ink preparation process, where a methyl ethyl ketone (MEK)–based P(VDF‐TrFE) solution is mixed with an isopropanol (IPA)–based 2D CIPS ink to form a uniform multicomponent dispersion. (b) Photographs of the resulting hybrid solution. (c) Crystal structure of ferroelectric CIPS. (d) Schematic illustration of the synergistic ferroelectric–ferroelectric coupling between CIPS and P(VDF‐TrFE), highlighting cooperative dipole alignment at the hybrid interface. (e) XRD patterns of pristine P(VDF‐TrFE) and P(VDF‐TrFE)/CIPS hybrid films with varying CIPS contents. (f) 3D illustration of the P(VDF‐TrFE)/CIPS film, showing the distribution of 2D CIPS nanosheets within the P(VDF‐TrFE) matrix.

To enhance the ferroelectricity of P(VDF‐TrFE), hybrid polymer solutions were prepared by incorporating an isopropanol (IPA)–based CuInP_2_S_6_ (CIPS) ink into a methyl ethyl ketone (MEK)‐based P(VDF‐TrFE) solution, as schematically illustrated in Figure 1a. The crystal structure of CIPS was characterized by XRD (Figure S1). Three main diffraction peaks appear at 2θ = 13.5°, 27.3°, and 56.4°, which can be indexed to the (002), (004), and (−335) planes of CIPS, respectively [40]. Liquid‐phase exfoliation of CIPS crystals synthesized via chemical vapor transport yields CIPS nanosheets with a thickness of approximately 10 nm (Figure S2). MEK was selected because it is fully miscible with IPA and has a comparable boiling point, which enables homogeneous mixing and simultaneous thermal annealing during solvent evaporation. The comparable boiling points and favorable miscibility between the two solvents facilitate uniform film formation characterized by a homogeneous distribution of 2D CIPS nanosheets within the polymer matrix. To systematically examine the effect of CIPS incorporation, the hybrid solutions were labelled PV‐CIPS‐03, −10, −15, and −22, corresponding to final (i.e., post‐mixing) CIPS concentrations of 0.03, 0.10, 0.15, and 0.22 mg mL^−1^, respectively (Figure 1b). These compositions were obtained by adding 0.1, 0.4, 0.75, and 1.5 mL of a 0.4 mg mL^−1^ CIPS ink to a 6 wt.% P(VDF‐TrFE) solution, thereby enabling precise control over the filler content while maintaining uniform solution processing conditions. Increasing the CIPS content required the use of proportionally larger volumes of the CIPS ink. Such higher 2D filler loadings were achieved by decreasing the polymer fraction in the P(VDF‐TrFE)/CIPS mixtures (see Table S1). Films prepared from these hybrid solutions are hereafter referred to as PV‐CIPS‐XX films when the CIPS concentration needs to be specified. Otherwise, they are described as P(VDF‐TrFE)/CIPS films. Atomic force microscopy (AFM) was used to measure the thicknesses of pristine P(VDF‐TrFE) and P(VDF‐TrFE)/CIPS films. The average thickness decreased from ∼700 nm for pristine P(VDF‐TrFE) to 510, 300, and 220 nm for PV‐CIPS‐03, −10, and −15, respectively (see Figure S3). This behavior is consistent with the concentration‐dependent thinning reported for P(VDF‐TrFE) films [41]. At the highest CIPS content (PV‐CIPS‐22), the film exhibited a non‐uniform and island‐like morphology as a result of the limited solubility of P(VDF‐TrFE) in IPA, and was therefore excluded from AFM investigation. For the remaining compositions, AFM topography reveals that CIPS incorporation leads to very smooth polymer films, with PV‐CIPS‐03 and PV‐CIPS‐10 exhibiting reduced root‐mean square roughness (R_RMS_) compared to pristine P(VDF‐TrFE) (Figure S4). In terms of the full width at half maximum of the ferroelectric (200) reflections, obtained from XRD analyses (Figure 1e), all three examined samples exhibit comparable values (Figure S5), indicating that their crystal quality remains similar. In contrast, PV‐CIPS‐15 and ‐22 display reduced (200) intensities, consistent with the morphological non‐uniformity observed by AFM (Figure S3). PV‐CIPS‐10 shows the strongest CIPS (002) reflection, indicating a strong c‐axis preferred orientation arising from the horizontal alignment of 2D CIPS flakes on the substrate. This configuration allows the intrinsic out‐of‐plane ferroelectric polarization of 2D CIPS to effectively couple with the dipoles of the P(VDF‐TrFE) matrix, thereby reinforcing the overall ferroelectric response of the hybrid film. Notably, although PV‐CIPS‐03 contains less CIPS, its significant enhancement in the P(VDF‐TrFE) (200) peak demonstrates that even minimal CIPS loading can promote β‐phase formation, underscoring a key advantage of the P(VDF‐TrFE)/CIPS hybrid strategy. Figure 1f illustrates the structural configuration of the hybrid P(VDF‐TrFE)/CIPS film, highlighting the parallel alignment of the CIPS nanosheets relative to the Au electrodes. As shown in Figure 1c,d, the out‐of‐axis dipoles of P(VDF‐TrFE) couple synergistically with the out‐of‐plane polarization of the CIPS nanosheets, leading to cooperative dipole alignment. The intrinsic polarization of CIPS generates a local electrostatic field that interacts with the dipoles of adjacent P(VDF‐TrFE) chains, promoting polarization locking and stabilizing the ferroelectric dipole configuration [42, 43].

**FIGURE 2:**
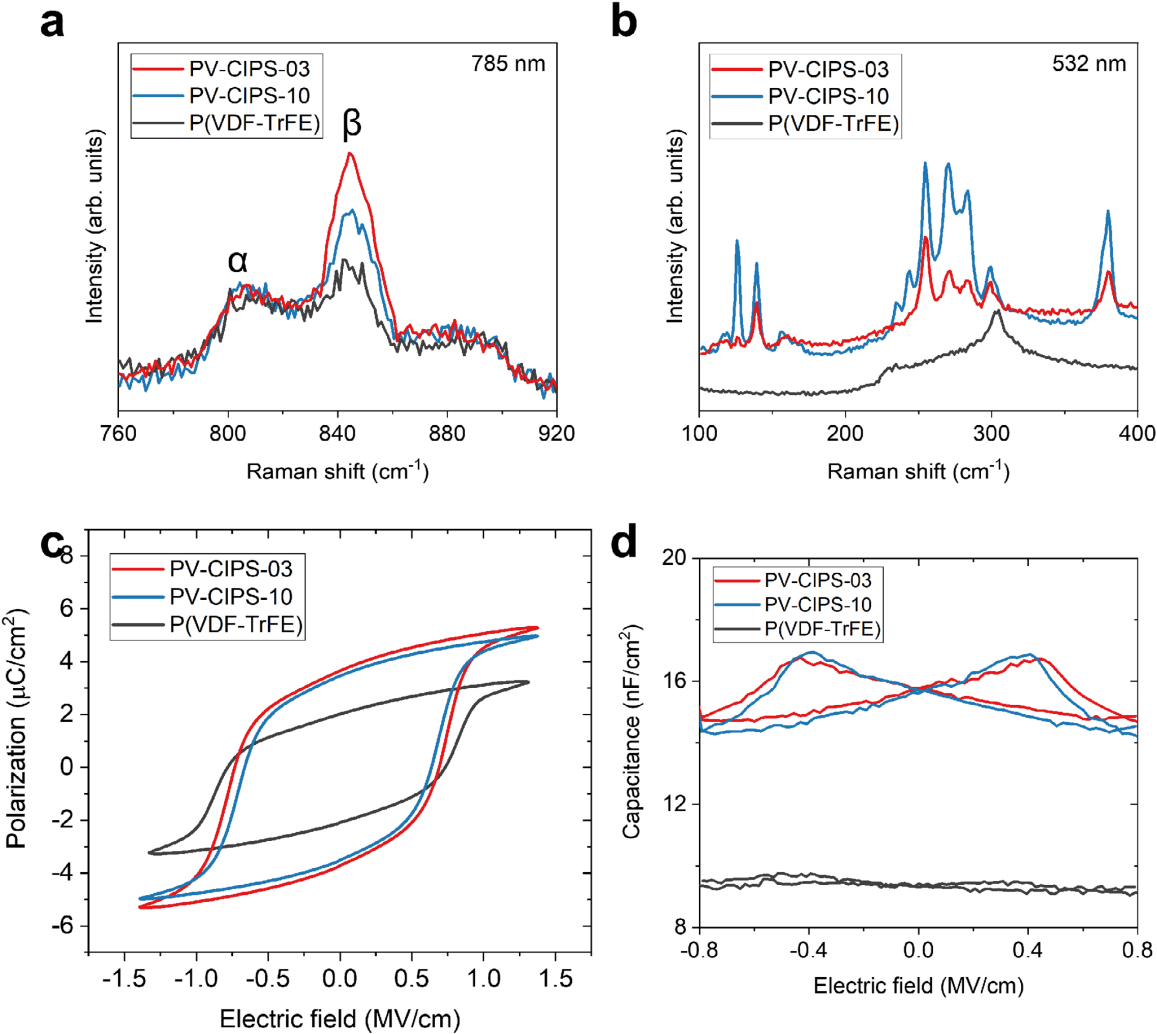
Raman and electrical characterizations of P(VDF‐TrFE)/CIPS hybrid films. (a, b) Raman spectra of pristine P(VDF‐TrFE), PV‐CIPS‐03, and PV‐CIPS‐10 films under 785 nm (a) and 532 nm (b) laser excitation. (c) Polarization–electric field (P‐E) hysteresis loops of Au/P(VDF‐TrFE)/Au, Au/PV‐CIPS‐03/Au, and Au/PV‐CIPS‐10/Au capacitors on SiO_2_ substrates, measured at 100 Hz. (d) C‐V characteristics of the same capacitor structures measured at 1 kHz.

The enhanced ferroelectricity inferred from the XRD analysis is further corroborated by Raman spectroscopy. As shown in Figure 2a, the P(VDF‐TrFE)/CIPS hybrid films exhibit a noticeably stronger β‐phase P(VDF‐TrFE) Raman peak at ∼845 cm^−1^ relative to the α‐phase peak at ∼805 cm^−1^, compared with pristine P(VDF‐TrFE) [44]. PV‐CIPS‐03 displays an even higher β‐phase intensity than PV‐CIPS‐10, indicating that a small amount of CIPS is sufficient to promote β‐phase crystallization. Under 532 nm excitation, characteristic Raman modes of 2D CIPS appear distinctly in the hybrid films (Figure 2b), confirming the successful incorporation of CIPS nanosheets into the P(VDF‐TrFE) matrix [45, 46]. To quantitatively assess the ferroelectric performance, metal–insulator–metal capacitors with Au/P(VDF‐TrFE)/Au and Au/P(VDF‐TrFE)‐CIPS/Au structures were fabricated for *P–E* measurements (Figure 2c). The pristine P(VDF‐TrFE) film exhibits a remnant polarization (*P*_r_) of ∼2.02 µC cm^−2^, whereas PV‐CIPS‐03 and PV‐CIPS‐10 show markedly higher values of ∼3.67 and ∼3.47 µC cm^−2^, corresponding to an enhancement of ∼75%. Concurrently, *E*_c_ decreases from ∼0.75 MV cm^−1^ for pristine P(VDF‐TrFE) to ∼0.69 MV cm^−1^ (PV‐CIPS‐03) and ∼0.64 MV cm^−1^ (PV‐CIPS‐10), reflecting facilitated dipole switching in the hybrid films. Together, these electrical characteristics indicate strong ferroelectric–ferroelectric coupling between the P(VDF‐TrFE) chains and the embedded CIPS nanosheets. Capacitance–voltage (*C*–*V*) measurements further highlight improved dielectric behavior in the hybrid films (Figure 2d). P(VDF‐TrFE)/CIPS devices exhibit clear ferroelectric hysteresis, consistent with reversible polarization switching. Moreover, the average capacitance within ±0.8 MV cm^−1^ increases to ∼15.5 nF cm^−2^ for P(VDF‐TrFE)/CIPS films, compared with ∼9.3 nF cm^−2^ for pristine P(VDF‐TrFE), representing an enhancement of ∼66%. This trend aligns with previous reports showing that the introduction of a small volume fraction of functional fillers can increase the dielectric constant of polymer ferroelectrics [47, 48]. Dielectric constant values for each film are summarized in Table S1. Overall, these results confirm that the incorporation of ultrathin 2D CIPS nanosheets into the P(VDF‐TrFE) yields a synergistic improvement in ferroelectric functionality (e.g., enhanced *P*_r_, reduced *E*_c_, and increased dielectric constant), with PV‐CIPS‐03 exhibiting the best balance.

**FIGURE 3:**
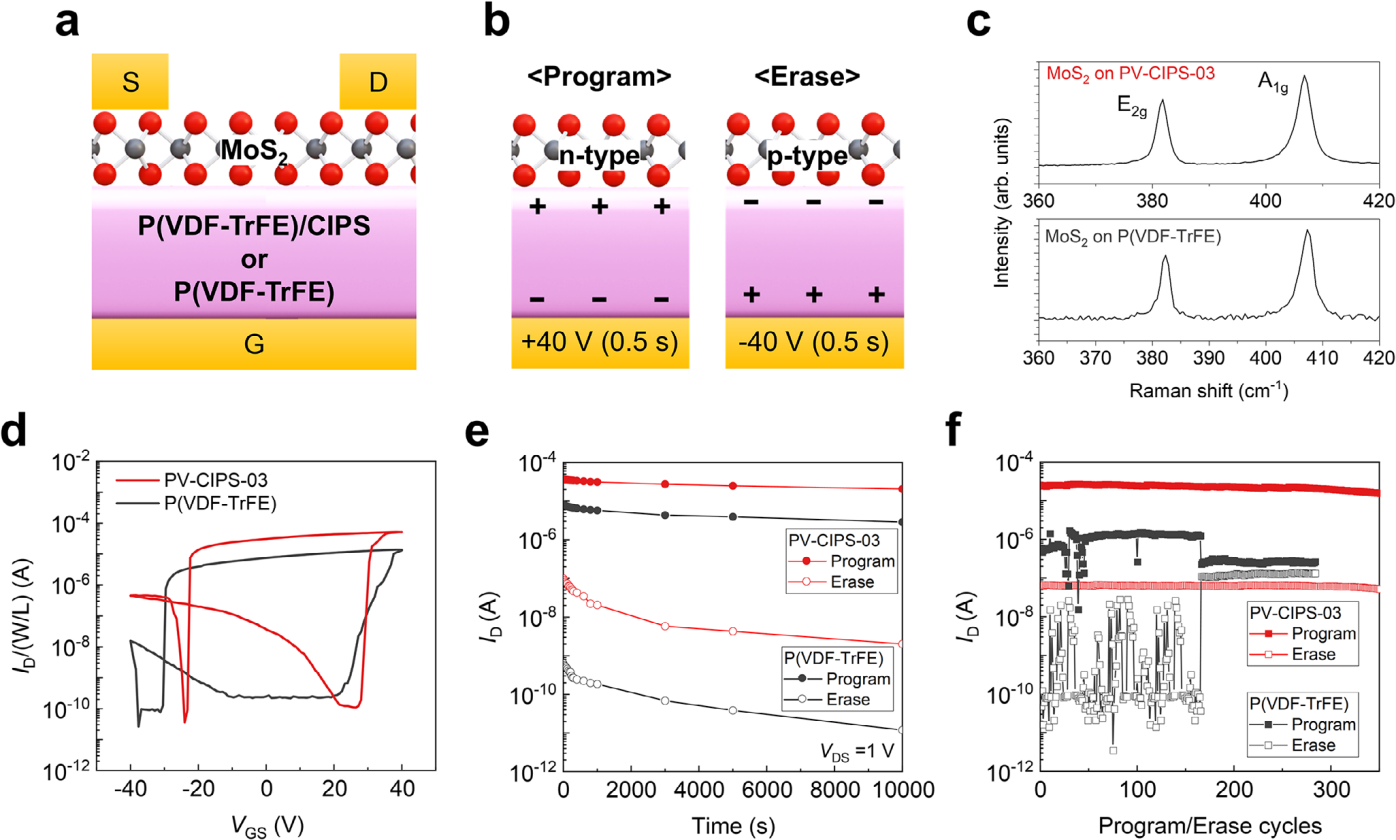
Electrical characterizations of MoS_2_‐based ferroelectric memory transistors incorporating PV‐CIPS‐03. (a) 2D illustration of the MoS_2_‐based ferroelectric memory transistor. (b) Schematics depicting two nonvolatile polarization states in the device: Program (electron‐accumulation) state and Erase (hole‐accumulation) state. (c) Raman spectra of MoS_2_ on PV‐CIPS‐03 (red) and P(VDF‐TrFE) (black). (d) Transfer characteristics, (e) retention properties, and (f) cyclic endurance results of the MoS_2_/PV‐CIPS‐03 FET (red) and the MoS_2_/P(VDF‐TrFE) FET (black) measured at *V*_DS_ = 1 V.

Among the P(VDF‐TrFE)/CIPS hybrids, PV‐CIPS‐03, which exhibits the highest *P*_r_, was selected as the gate dielectric for fabricating MoS_2_‐based ferroelectric memory transistors. Figure 3a shows the device architecture composed of a MoS_2_ channel, a PV‐CIPS‐03 gate insulator, and an Au gate electrode on SiO_2_/n^+^ Si substrates, with Au also serving as the source and drain contacts. Detailed fabrication procedures are provided in the Experimental Section and Figure S6. For nonvolatile operation, the electron accumulated n‐type conduction state is defined as the Program state, and the hole accumulated p‐type conduction state is defined as the Erase state. These states correspond to the conductivity levels of MoS_2_ after applying gate voltage (*V*_GS_) pulses of +40 V for 0.5 s and −40 V for 0.5 s, respectively, as illustrated in Figure 3b. Raman spectra in Figure 3c, collected from MoS_2_ supported on PV‐CIPS‐03 and on pristine P(VDF‐TrFE), confirm that the MoS_2_ lattice remains intact despite the roughness of the underlying polymer films [49]. Based on the frequency separation and intensity ratio of the E_2g_ and A_1g_ modes, the MoS_2_ channels in both devices consist of approximately four to seven layers in thickness [49]. This thickness range was chosen to balance carrier transport and electrostatic control, enabling more efficient ambipolar behavior compared to monolayer MoS_2_. Figure 3d compares the transfer characteristics of the MoS_2_/PV‐CIPS‐03 and MoS_2_/P(VDF‐TrFE) transistors. At *V*_GS_ = +40 V, the normalized drain current (*I*_D_) increases from 12.7 µA in the P(VDF‐TrFE) device to 51.3 µA in the PV‐CIPS‐03 device. This increase, which corresponds to approximately a factor of 4, indicates stronger electron accumulation. At *V*_GS_ = −40 V, the hole current increases from 14.5 to 381.8 nA, corresponding to a factor of 26. This behavior clearly demonstrates efficient hole carrier accumulation in MoS_2_, which is rarely observed in MoS_2_ based ferroelectric transistors that use pristine P(VDF‐TrFE) as the dielectric [50]. Near *V*_GS_ = 0 V, the mobility trends are consistent with these observations. The electron mobility increases from 11.83 to 44.36 cm^2^ V^−1^ s^−1^, and the hole mobility increases from 0.0012 to 0.3485 cm^2^ V^−1^ s^−1^. The 300‐fold increase in the hole mobility is evidenced in the linear mobility versus *V*_GS_ plots presented in Figure S7. The hysteresis window decreases from 60 to 52 V in the PV‐CIPS‐03 device. This reduction agrees with the smaller *E*_c_ observed in the P‐E measurements of Figure 2c. These results confirm that the ferroelectric reinforcement observed in metal‐insulator‐metal capacitors is preserved when the hybrid film is used as a gate dielectric to support a 2D semiconductor channel. Importantly, the P(VDF‐TrFE)/CIPS dielectric enables polarization‐driven dipole alignment at lower operating voltages than pristine P(VDF‐TrFE), suggesting that further optimization of hybrid film thickness and capacitance could enable low‐voltage operation of 2D ferroelectric memory transistors. In addition, thickness‐dependent scaling behavior of the ferroelectric properties has been systematically investigated and is presented in the Supporting Information (Figures S8–S10), where the variation of *E*_c_ and coercive voltage with film thickness is analyzed [51, 52]. While interfacial effects become more pronounced as the film thickness decreases, the reduced thickness is still expected to enable low‐voltage operation of 2D electronic devices. Significantly, in our MoS_2_/PV‐CIPS‐03 device, the negative *V*_GS_ pulse not only suppresses electron transport but also induces considerable hole doping in MoS_2_. This behavior has not been observed in MoS_2_/P(VDF‐TrFE) control devices, as displayed in Figure 3d,e. Retention measurements in Figure 3e show that both the Program and Erase states of the PV‐CIPS‐03 device remain stable for approximately 10,000 s. The difference between PV‐CIPS‐03 and pristine P(VDF‐TrFE) becomes even more evident in endurance tests. Figure 3f shows that the MoS_2_/P(VDF‐TrFE) device exhibits large fluctuations, particularly in the Erase state, and fails after approximately one hundred sixty cycles. On the other hand, the MoS_2_/PV‐CIPS‐03 device retains stable Program and Erase states for over three hundred cycles. These results highlight the superior operational stability and endurance enabled by the P(VDF‐TrFE)/CIPS hybrid dielectric. Meanwhile, PV‐CIPS‐10 devices exhibit weaker retention, confirming PV‐CIPS‐03 as the optimal composition (Figure S11).

**FIGURE 4:**
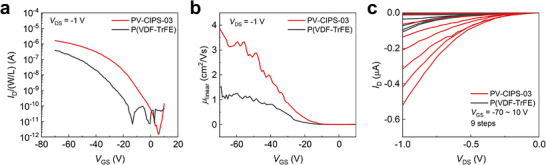
Electrical characterizations of unipolar p‐type transistor operation in MoS_2_/PV‐CIPS‐03 ferroelectric FET. (a) Transfer curves, (b) mobility plots, and (c) output curves of the MoS_2_/PV‐CIPS‐03 FET (red) compared with the MoS_2_/P(VDF‐TrFE) control device (black) at *V*_DS_ = −1 V.

To further evaluate the unipolar p‐type performance suggested by the ambipolar ferroelectric memory characteristics, the *V*_GS_ range was extended to −70 V. Under this condition, the MoS_2_/PV‐CIPS‐03 transistor exhibits a maximum *I*_D_ of 1.6 µA at *V*_GS_ = −70 V, along with an *I*_ON_/*I*_OFF_ ratio of 10^6^. Using 10^−10^ A as the threshold‐current criterion, the extracted threshold voltage (*V*_TH_) amounts to 0 V. This represents a considerable positive shift relative to the control device that shows *V*_TH_ = −20 V, reflecting a substantially higher hole concentration in the MoS_2_ channel when employing the PV‐CIPS‐03 dielectric. This conclusion is further supported by the 4.2‐fold increase in the maximum *I*_D_​ of the PV‐CIPS‐03 transistor relative to the control sample at *V*_GS_ = −70 V. The hole linear mobility also improves from 1.5 to 3.9 cm^2^ V^−1^ s^−1^ (2.6‐fold enhancement). The achieved mobility of 3.9 cm^2^ V^−1^ s^−1^, though not the highest among all p‐type MoS_2_ FETs reported, stands out as one of the most effective results reported for devices employing organic interface functionalization, a field in which systematic demonstrations remain relatively scarce [50, 53]. Figure 4c displays the output characteristics of the MoS_2_/PV‐CIPS‐03 transistor, revealing a 5‐fold increase in the maximum *I*_D_ at *V*_GS_ = −70 V and *V*_DS_ = −1 V. The MoS_2_/Au contacts exhibit clear Schottky behavior for hole injection, consistent with the fact that Au forms more favorable near‐ohmic contacts for electron conduction but presents an energy barrier for hole transport into MoS_2_ [54, 55]. Output curves under both Program (n‐type) and Erase (p‐type) states are provided in Figure S12, highlighting the ohmic‐like n‐type contact and the Schottky p‐type contact behaviors.

**FIGURE 5:**
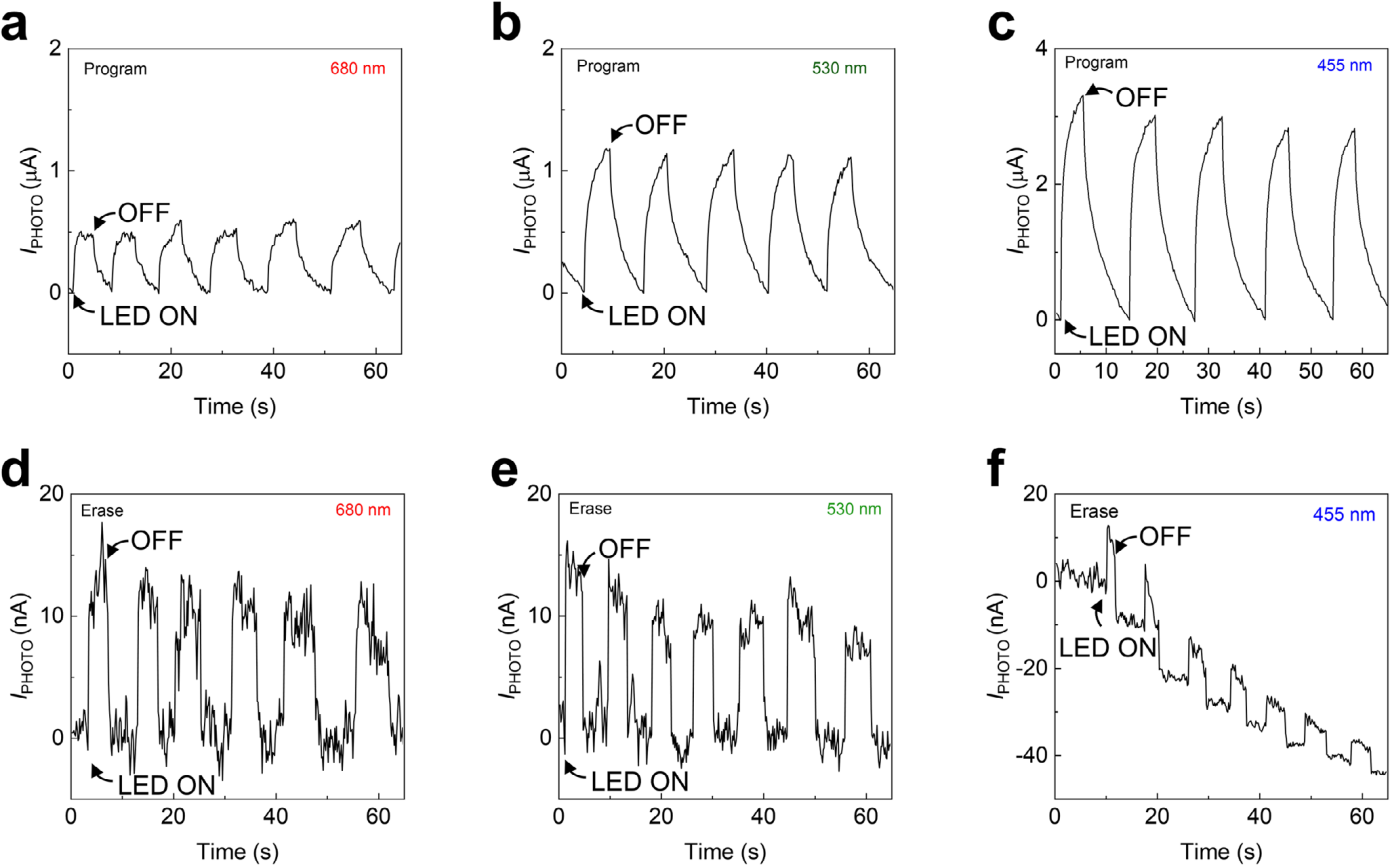
Photoresponse of MoS_2_/PV‐CIPS‐03 ferroelectric memory FET. (a–c) Time‐resolved *I*_PHOTO_ measured in the ferroelectric Program state under 680, 530, and 455 nm LED illumination. (d–f) Time‐resolved *I*_PHOTO_ measured in the ferroelectric Erase state under 680, 530, and 455 nm LED illumination. All measurements were performed at *V*_GS_ = 0 V and *V*_DS_ = 1 V.

Finally, we examined the photoresponse characteristics of the MoS_2_/PV‐CIPS‐03 transistor. Upon illumination with 680, 530, and 455 nm (intensity: 0.5 mW cm^−2^) LEDs, the transistor exhibits high responsivities in the Program state, where electrons are accumulated (Figure 5a–c). The estimated values are 4,000 A W^−1^ (680 nm), 12,000 A W^−1^ (530 nm), and 26,400 A W^−1^ (455 nm). In contrast, the Erase state yields lower responsivity of ∼800 A W^−1^ across all three wavelengths, but demonstrates notably faster photo‐switching dynamics (Figure 5d–f). For both Program and Erase states, stable photoresponses are observed, indicating that ferroelectric polarization is largely preserved under optical excitation. The only exception occurs under 455 nm illumination in the Erase state. Under this condition, interestingly, the *I*_PHOTO_ gradually decreases over time. This decay is attributed to a progressive reduction in the ferroelectric polarization of CIPS under photoexcitation, in agreement with previous reports showing that CIPS polarization weakens under above‐bandgap illumination [56, 57]. Such gradual decreases in I_PHOTO_ are most evident in the low‐responsivity Erase state and suggest a potential route toward photon‐assisted negative photoconductivity and multilevel memory operation. Importantly, no photocurrent decay is observed in the MoS_2_/P(VDF‐TrFE) control transistor under identical 455 nm illumination in the Erase state. This contrast confirms that the observed depolarization arises from the CIPS nanosheets, which have a bandgap of 2.6–2.7 eV [58, 59]. The normal photoresponse of the control device under 455 nm illumination in the Erase state is provided in Figure S13, where the photocurrent is mainly attributed to photo‐excited electrons, consistent with previous reports [60, 61, 62, 63].

## 3 Conclusion

We have established a hybrid 2D material—polymer platform displaying significantly enhanced ferroelectricity. The uniform incorporation of 2D CuInP_2_S_6_ (CIPS) nanosheets into a P(VDF‐TrFE) matrix through a solvent‐balanced IPA–MEK solution process was demonstrated as a simple and scalable approach to produce homogeneous filler dispersion and to promote β‐phase crystallization of P(VDF‐TrFE), resulting overall in a synergistic enhancement of ferroelectric properties. The fine‐tuning of the relative contents of the two ferroelectric components enabled efficient cooperative dipole coupling between the polymer chains and the embedded CIPS nanosheets. MoS_2_‐based ferroelectric memory transistors utilizing PV‐CIPS‐03 as the gate dielectric exhibit pronounced ambipolar behavior, significantly strengthened electron and hole conduction, stable nonvolatile switching, and superior endurance compared with P(VDF‐TrFE)‐based transistors. Under extended negative gating, the hybrid dielectric further enables high‐performance unipolar p‐type operation, achieving one of the highest reported hole mobilities for MoS_2_ FETs via non‐destructive organic interface functionalization. In addition, the P(VDF‐TrFE)/CIPS layer imparts wavelength‐dependent photoresponse and unique photon‐driven ferroelectric modulation, suggesting new opportunities for negative photoconductivity and multilevel optoelectronic memory. By combining enhanced ferroelectricity, reliable memory operation, and optically addressable functionality within a solution‐processed hybrid film, this work positions CIPS‐integrated P(VDF‐TrFE) as a versatile materials platform for future flexible, low‐power, and multifunctional ferroelectric electronics.

## 4 Experimental Section

### 4.1 Materials

Bulk 2H‐MoS_2_ crystals were purchased from HQ Graphene. P(VDF‐TrFE), IPA, and 2‐butanone were purchased from Sigma‐Aldrich (Product codes: 900904 and 360473). Copper (99.9%) and indium (99.9%) powders were purchased from Alfa Aesar, Germany.

### 4.2 CuInP_2_S_6_ Crystal Preparation

A stoichiometric mixture of copper, indium, phosphorus, and sulfur, equivalent to 15 g of thiophosphite, was loaded into a quartz glass ampoule (30 × 150 mm^2^, wall thickness: 3 mm) and hermetically sealed under high vacuum (<1 × 10^−3^ Pa) using an oxygen/hydrogen welding torch. The sealed ampoule was then placed in a muffle furnace, where the precursor mixture was subjected to heat treatment at 650°C for 120 h and then maintained at 700°C for an additional 120 h. Both the heating and cooling processes were carried out at a controlled rate of 1°C min^−1^. The ampoule was subsequently placed in a gradient two‐zone furnace. First, the growth zone was heated to 750°C and the source zone to 600°C. After two days, the thermal gradient was reversed, and the source zone was heated to 750°C and the growth zone to 650°C for 14 days. After cooling to room temperature, the ampoule was opened in an argon‐filled glovebox, and the grown crystals were collected.

### 4.3 CuInP_2_S_6_ Ink Preparation

Initially, 0.5 g of CIPS powder was combined with 1.5 mL of IPA and milled in a mortar for about 5 min. After evaporation of the grinding solvent, an additional 1.5 mL of IPA was added, and the mixture was further processed. Subsequently, 100 mg of the ground material was transferred into 10 mL of fresh IPA to form a suspension, which was exposed to bath ultrasonication (∼90 kHz) for 2 h. The obtained dispersion was then clarified by centrifugation at 150 g (Eppendorf 5804) for 30 min. The upper 3/4 of the supernatant was carefully decanted and centrifuged again under the same conditions. Finally, the top 2/3 of the supernatant from this second step was collected for subsequent analyses.

### 4.4 Device Fabrication—Metal‐Insulator‐Metal (MIM) Capacitors and Structural Characterization

For structural and ferroelectric characterization, P(VDF‐TrFE) and PV‐CIPS‐XX films were prepared for XRD, Raman, and AFM measurements. The polymer and hybrid films were spin‐coated onto SiO_2_ substrates under ambient conditions and subsequently annealed in a nitrogen (N_2_) atmosphere. For *P*–*E* and *C*–*V* measurements, MIM capacitors were fabricated using thermally evaporated Au as both bottom and top electrodes, where the polymer layers were spin‐coated under ambient conditions and annealed in a N_2_ atmosphere. For thickness‐dependent studies, all PV‐CIPS films used for AFM and *P*–*E* measurements were fully prepared under a N_2_ atmosphere to improve surface uniformity and ferroelectric properties.

### 4.5 Device Fabrication—MoS_2_/PV‐CIPS Ferroelectric Memory Transistors

The Au/Cr (12 nm/3 nm) bottom gate electrodes were patterned on the SiO_2_/n^+^‐Si substrates using photolithography (laser writer LW405B from Microtech, AZ1505 photoresist), thermal evaporation, and a lift‐off process. P(VDF‐TrFE) was dissolved in 2‐butanone to prepare a 6 wt.% solution, which was mixed with CuInP_2_S_6_ ink at various volume ratios to produce PV‐CIPS hybrids. The PV‐CIPS films were spin‐coated onto the bottom gate electrodes and annealed on a hot plate at 100°C for 1 min under ambient conditions. The semiconducting MoS_2_ channel was then transferred onto the PV‐CIPS/Au layers using the PDMS stamping method, also under ambient conditions. This was followed by curing at 142 °C on a hot plate for 2 h in N_2_ to crystallize the P(VDF‐TrFE). Finally, S/D electrodes of Au (50 nm) were patterned using the same methods as for the bottom electrode. The prepared devices were annealed at 100°C for 2 h in N_2_ to improve contact quality.

### 4.6 Optoelectronic Measurements

Electrical measurements were performed using a Keithley dual‐channel 2636A and 4200A SCS semiconductor parameter analyzer under ambient atmosphere and dark conditions. The capacitances of dielectric materials were measured by a precision LCR meter (Agilent 4284A) under the same conditions. The polarization‐electric field characteristic was measured by a ferroelectric tester (TF analyzer 1000) at 100 Hz and room temperature, with a top‐electrode area of 0.4 mm^2^. Photoresponse measurements of the transistors were conducted under the illumination of 680, 530, and 455 nm LEDs.

### 4.7 Material Characterization—Atomic Force Microscopy (AFM)

For AFM imaging, scan‐assist mode atomic force microscopy was performed in air using a Bruker Dimension Icon. The tip model was SCAN‐ASSIST‐AIR with a stiffness constant *K* = 0.4 N m^−1^. Surface morphology of films processed under a N_2_ atmosphere was characterized by atomic force microscopy (FX40, Park Systems) in non‐contact mode under ambient conditions using a PPP‐NCHR cantilever (NANOSENSORS, resonance frequency 330 kHz, spring constant 42 N m^−1^, tip radius < 10 nm). Topographic images were acquired at a fixed scan rate of 0.8 Hz.

### 4.8 Material Characterization—Confocal Raman Microscopy

Confocal Raman microscopy was carried out using a Renishaw InVia Qontor confocal microRaman system equipped with a front‐illuminated CCD camera and a research‐grade Leica DM 2700 microscope. Spectra were collected by using a 100× and a laser operating at 532 nm and 785 nm. Spectra were processed with the Renishaw software WiRE 5.5, and corrected for cosmic rays, baseline, and noise.

### 4.9 Material Characterization—X‐Ray Diffraction (XRD) Analysis

The crystallinity of ferroelectric P(VDF‐TrFE) polymer was investigated by X‐ray diffraction patterns (Bruker D8 x‐ray diffractometer).

## Author Contributions

Y.J. and H.W. contributed equally to this work. Y.J., H.W., and P.S. conceived the experiments and designed the study. Y.J. and H.W. performed all optoelectrical and materials characterization. H.W. prepared the CuInP_2_S_6_ ink. P.T. performed the Raman measurements and data analysis. A.T. and H.S.A performed the AFM measurements. B.W. and Z.S. synthesized the CuInP_2_S_6_ crystals. Y.C.H. performed the P‐E measurements. Y.J., H.W., P.T., A.T., B.W., Z.S., Y.C.H., J.H.J., M.B. and P.S. discussed the results and contributed to data interpretation. Y.J., H.W., and P.S. co‐wrote the paper with input from all co‐authors.

## Conflicts of Interest

The authors declare no conflict of interest.

## Supporting information




**Supporting File**: advs76127‐sup‐0001‐SuppMat.docx.

## Data Availability

The data that support the findings of this study are available from the corresponding author upon reasonable request.
