# Supplementary material for: Boosting Ferroelectricity: 2D and Polymer Ferroelectric Hybrids Enabling Ambipolar Nonvolatile MoS2 Memory Transistor
Source: Adv Sci (Weinh). 2026 Jun 15:e76127. Online ahead of print. doi: 10.1002/advs.76127 (PMC13337029; doi:10.1002/advs.76127)
Supplement: Supplementary file 1 — Supporting File: advs76127‐sup‐0001‐SuppMat.docx. [file ADVS-9999-e76127-s001.docx]

Supporting Information

**Boosting Ferroelectricity: 2D and Polymer Ferroelectric Hybrids Enabling Ambipolar Nonvolatile MoS_2_ Memory Transistor**

*Yeonsu Jeong,^#^ Honglei Wang,^#^ Pietro Tordi, Ying Chieh Hu, Adrián Tamayo, Bing Wu, Hyun Soo Ahn, Zdenek Sofer, Jong Hoon Jung, Massimo Bonini,* Paolo Samorì**

Dr. Honglei Wang, Dr. P. Tordi, Dr. A. Tamayo, Prof. Dr. P. Samorì

University of Strasbourg & CNRS, ISIS & icFRC, 8 allee Gaspard Monge, 67000 Strasbourg, France.

Email: [samori@unistra.fr](mailto:samori@unistra.fr)

Dr. B. Wu, Prof. Dr. Zdenek Sofer

Department of Inorganic Chemistry, University of Chemistry and Technology Prague, Technická 5, Prague 6 166 28, Czech Republic

Prof. Dr. Y. Jeong, Y. C. Hu, H. S. Ahn, Prof. Dr. J. H. Jung

Institute of Quantum Science, Department of Physics, Inha University, 100 Inha-ro, Michuhol-gu, Incheon, 22212, Republic of Korea

H. S. Ahn, Prof. Dr. J. H. Jung

Program in Semiconductor and Device, Inha University, 100 Inha-ro, Michuhol-gu, Incheon, 22212, Republic of Korea

Dr. P. Tordi, Prof. Dr. M. Bonini

Department of Chemistry “Ugo Schiff” and CSGI, University of Florence, via della Lastruccia 3, Sesto Fiorentino, Florence, 50019 Italy

Email: massimo.bonini@unifi.it

*^#^* These authors contributed equally to this work

**Keywords:** MoS_2_, CuInP_2_S_6_, P(VDF-TrFE), hybrid ferroelectrics, ambipolar memory

**Table of Contents**

**Table S1.** General properties of CuInP_2_S_6_ (CIPS)/P(VDF-TrFE) hybrids

**Figure S1.** XRD patterns of CIPS crystal

**Figure S2.** Topographical AFM image of CIPS nanosheets.

**Figure S3.** Thickness profiles of pristine P(VDF-TrFE) and P(VDF-TrFE)/CIPS hybrid films with varying CIPS contents.

**Figure S4.** Topographical AFM image of pristine P(VDF-TrFE), PV-CIPS-03, and PV-CIPS-10.

**Figure S5.** Enlarged XRD profiles of the (200) ferroelectric peak for pristine P(VDF-TrFE), PV-CIPS-03, and PV-CIPS-10 hybrid films.

**Figure S6.** Fabrication process of MoS_2_-based ferroelectric memory transistors.

**Figure S7.** Optical microscopy images, and linear mobility plots of MoS_2_/PV-CIPS-03 and control devices.

**Figure S8.** Thickness-dependent ferroelectric properties of PV-CIPS films.

**Figure S9.** Film thickness of PV-CIPS-03 and PV-CIPS-10 as a function of spin coating speed with the corresponding root-mean-square roughness (R_RMS_).

**Figure S10.** Topographical AFM image of PV-CIPS-03 and PV-CIPS-10 as a function of spin coating speed.

**Figure S11.** Electrical characteristics of MoS_2_/PV-CIPS-03 and MoS_2_/PV-CIPS-10 ferroelectric memory devices.

**Figure S12.** Output characteristics of the MoS_2_/PV-CIPS-03 device under the Program (n-type) state and the Erase (p-type) state.

**Figure S13.** Photocurrent response of the MoS_2_/P(VDF-TrFE) control device under 455 nm illumination in the erase state.

**Table S1.** Solution composition, film thickness, surface roughness, XRD (200) peak characteristics, and electrical properties of CuInP_2_S_6_/P(VDF-TrFE) hybrid films.

| **Sample** | **6 wt%**  **P(VDF-TrFE)**  **in MEK (ml)** | **0.05 wt%**  **CIPS in**  **IPA (ml)** | **CIPS in mixture (mg/ml)** | **Thickness (nm)** | **R_RMS_**  **(nm)** | **XRD (200)**  **peak intensity** | **FWHM**  **(200) peak** | ***P*_r_**  **(μC cm^-2^)** | ***C*_avg_ (nF cm^-2^)** | **Dielectric constant** |
| --- | --- | --- | --- | --- | --- | --- | --- | --- | --- | --- |
| **P(VDF-TrFE)** | **1.17** | **None** | **None** | **700** | **35.13** | **1.32** | **0.816** | **2.02** | **9.3** | **7.35** |
| **PV-CIPS-03** | **1.17** | **0.1** | **0.03** | **510** | **25.49** | **3.67** | **0.832** | **3.67** | **15.6** | **8.47** |
| **PV-CIPS-10** | **1.17** | **0.4** | **0.10** | **300** | **26.40** | **3.42** | **0.787** | **3.47** | **15.5** | **5.08** |
| **PV-CIPS-15** | **1.17** | **0.75** | **0.15** | **220** | **-** | **1.41** | **-** | **-** | **-** | **-** |
| **PV-CIPS-22** | **1.17** | **1.5** | **0.22** | **-** | **-** | **0.72** | **-** | **-** | **-** | **-** |

R_RMS_: root-mean-square roughness, FWHM: full width at half maximum


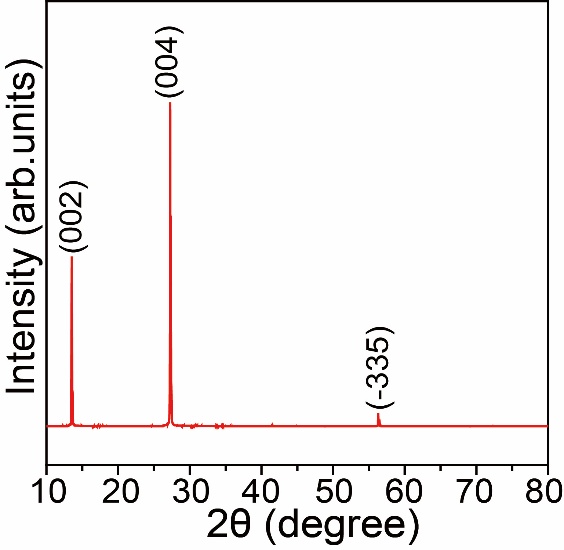


**Figure S1.** XRD patterns of CIPS crystal.


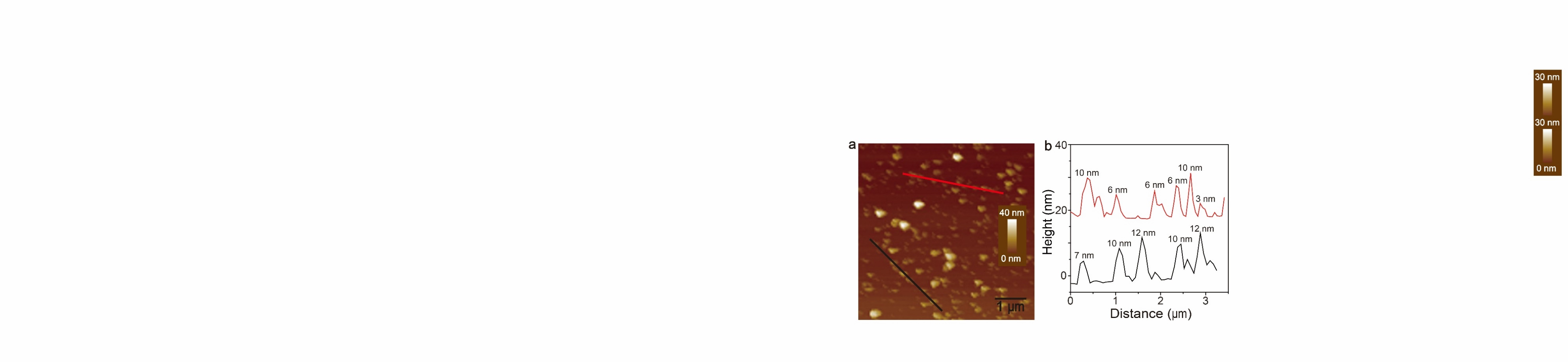
**Figure S2.** AFM topographical image of a) exfoliated CIPS nanosheets, and b) corresponding topographical profiles.


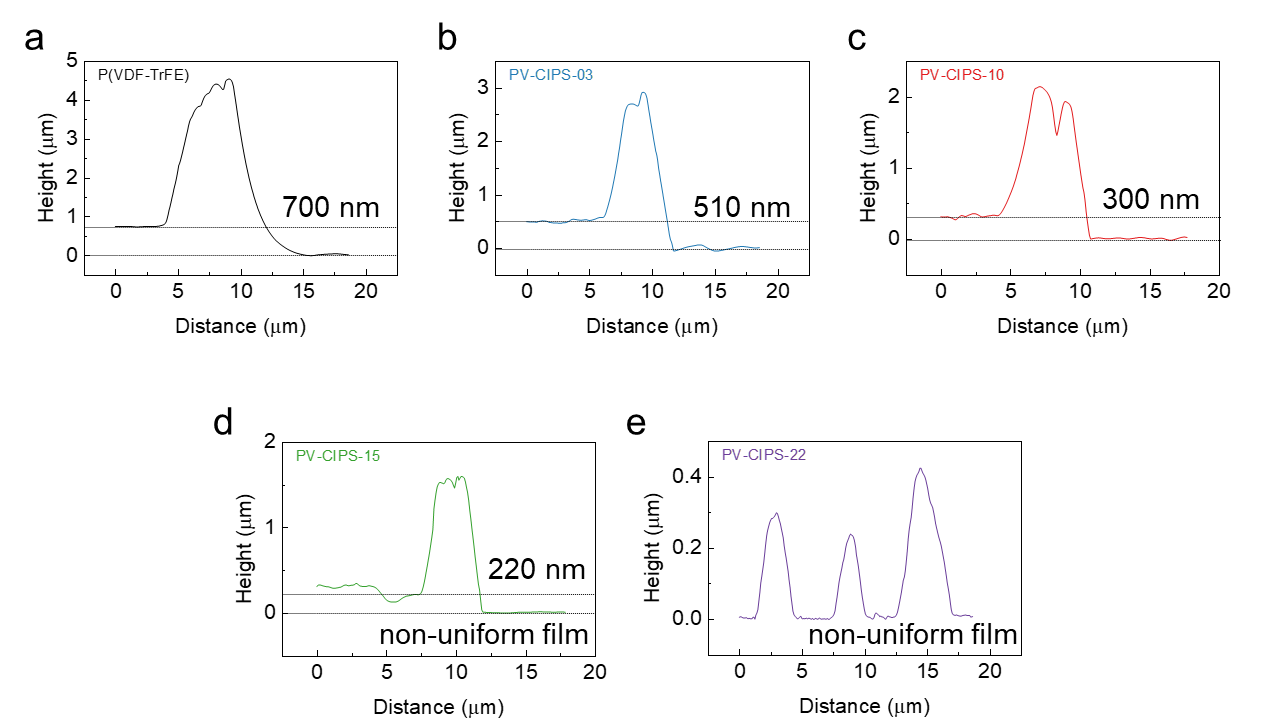


**Figure S3.** Thickness profiles, extracted from topographical AFM images, of a) pristine P(VDF-TrFE) and b-e) PV-CIPS hybrid films with varying CIPS contents.


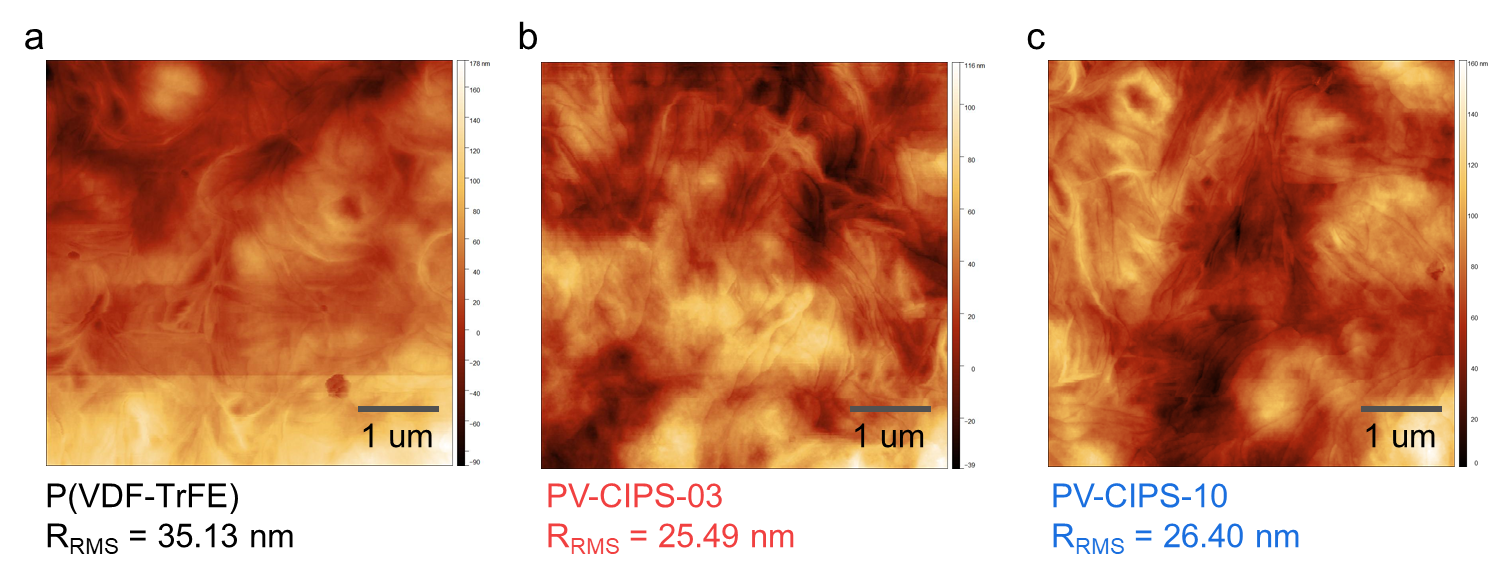


**Figure S4.** Topographical AFM image of a) pristine P(VDF-TrFE), b) PV-CIPS-03, and c) PV-CIPS-10, showing root-mean-square roughness (R_RMS_) of 35.13 nm, 25.49 nm, and 26.40 nm, respectively, as determined on a region of 5 μm $\times$ 5 μm. Such results reveal a clear smoothening effect upon CIPS incorporation into the P(VDF-TrFE) matrix.

**
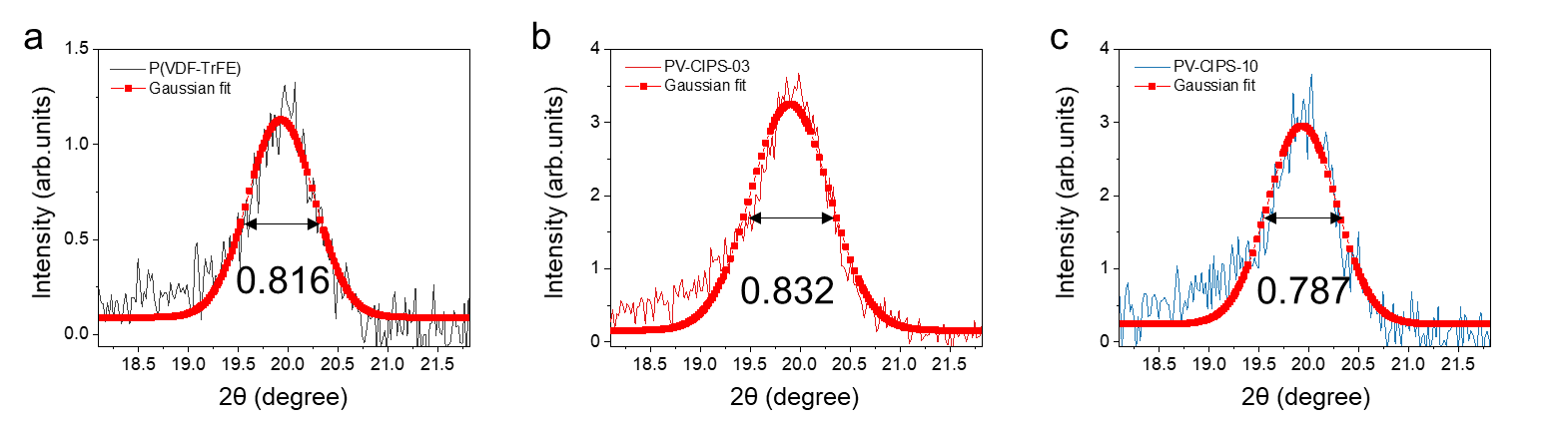
**

**Figure S5.** Enlarged XRD profiles of the (200) ferroelectric peak for a) pristine P(VDF-TrFE), b) PV-CIPS-03, and c) PV-CIPS-10 hybrid films. Each peak was fitted using a Gaussian function to extract the full width at half maximum (FWHM), yielding values of 0.816, 0.832, and 0.787, respectively.


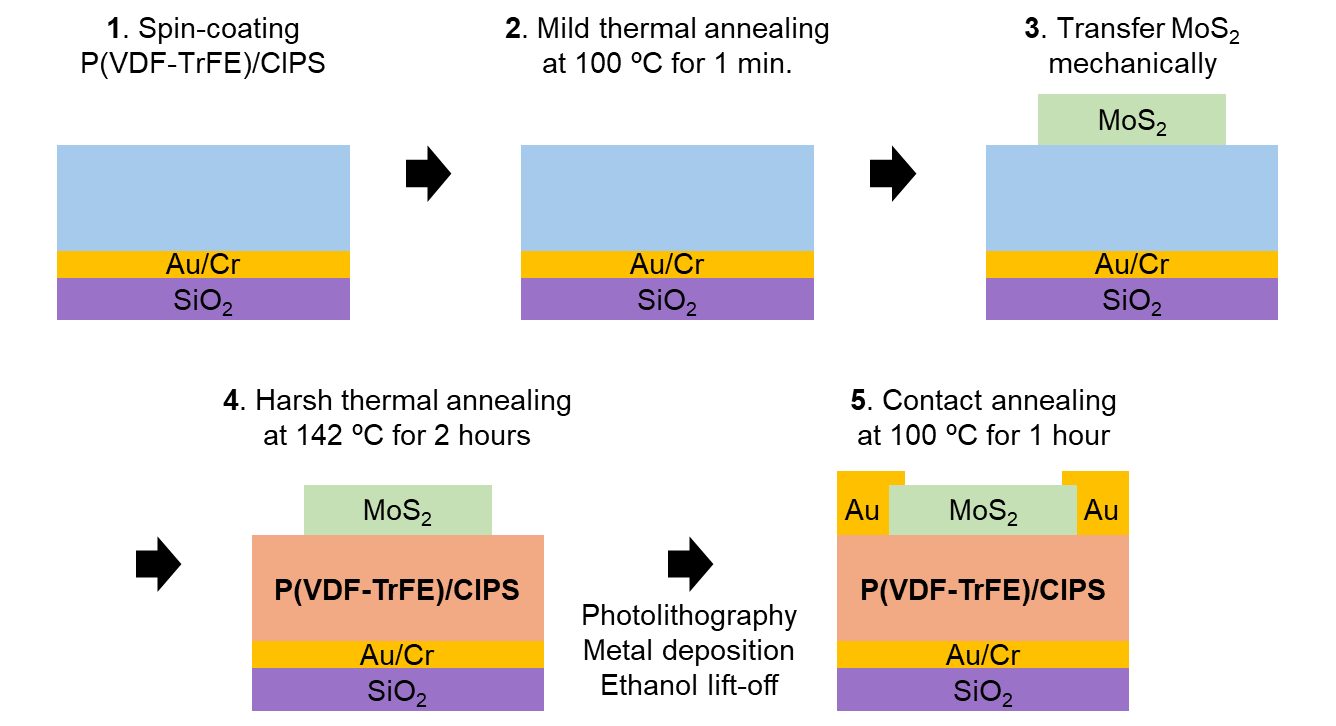


**Figure S6.** Fabrication process of MoS_2_-based ferroelectric memory transistors.


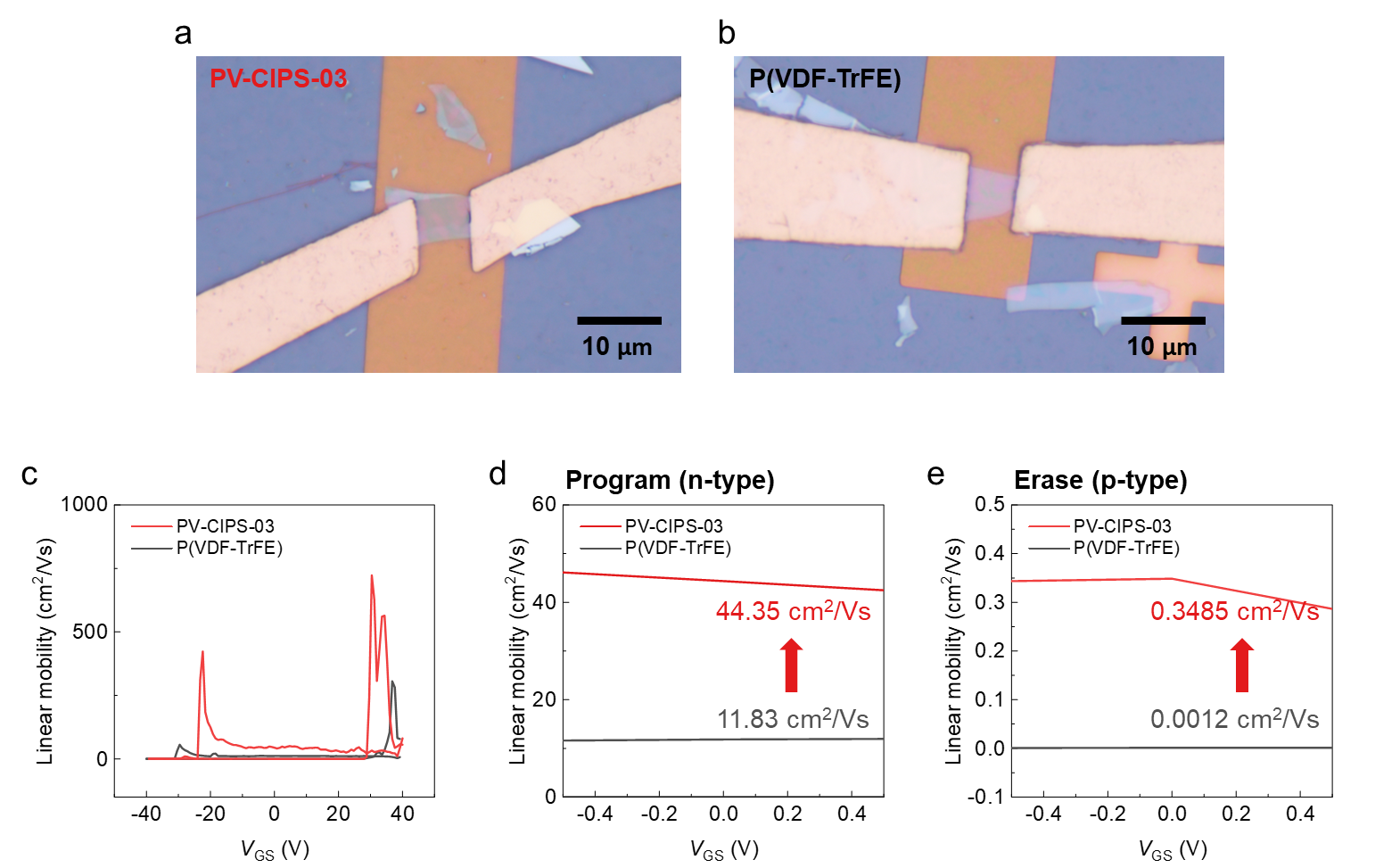


**Figure S7.** a, b) Optical microscopy images, and c) linear mobility plots of MoS_2_/PV-CIPS-03 and control devices. d) Extracted n-type mobility in the Program state, showing an increase from 11.83 cm^2^ V^-1^s^-1^ to 44.35 cm^2^ V^-1^s^-1^. e) Extracted p-type mobility in the Erase state, increasing from 0.0012 cm^2^ V^-1^s^-1^ to 0.3485 cm^2^ V^-1^s^-1^. These results confirm that CIPS incorporation significantly improves both n- and p-type transport characteristics of MoS_2_ channel.


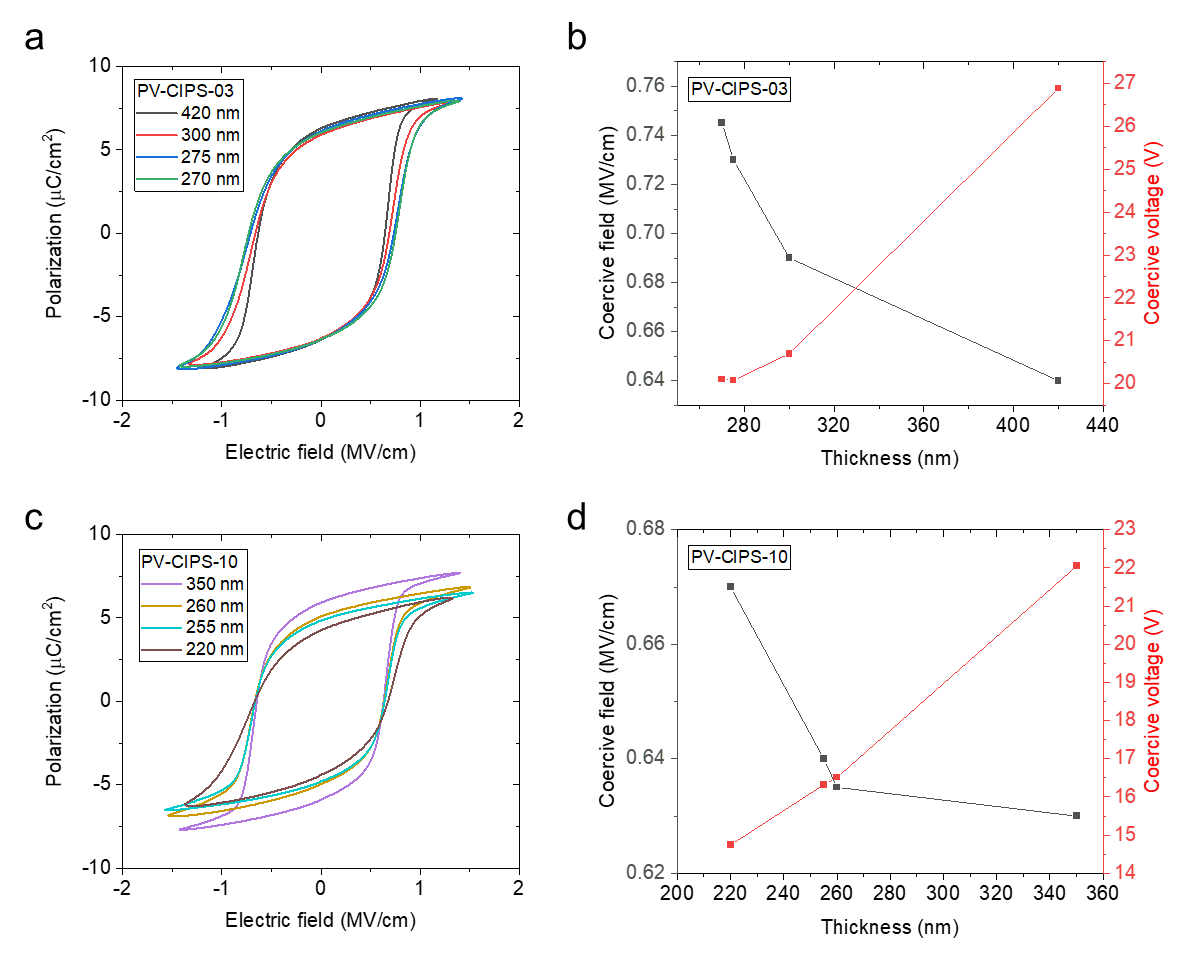


**Figure S8.** a,b) P–E hysteresis loops and extracted coercive field (*E*_c_) and coercive voltage (*V*_c_) of PV-CIPS-03 as a function of film thickness. The polarization behavior remains nearly unchanged across the thickness range. *E*_c_ increases with decreasing thickness, while *V*_c_ exhibits sub-linear scaling. c,d) *P–E* hysteresis loops and extracted *E*_c_ and *V*_c_ of PV-CIPS-10 as a function of film thickness. Compared to PV-CIPS-03, PV-CIPS-10 shows a weaker thickness dependence of *E*_c_. We attribute this result not to improved interfacial quality, but rather to increased nanosheet aggregation and reduced structural uniformity at higher CIPS loading.

**
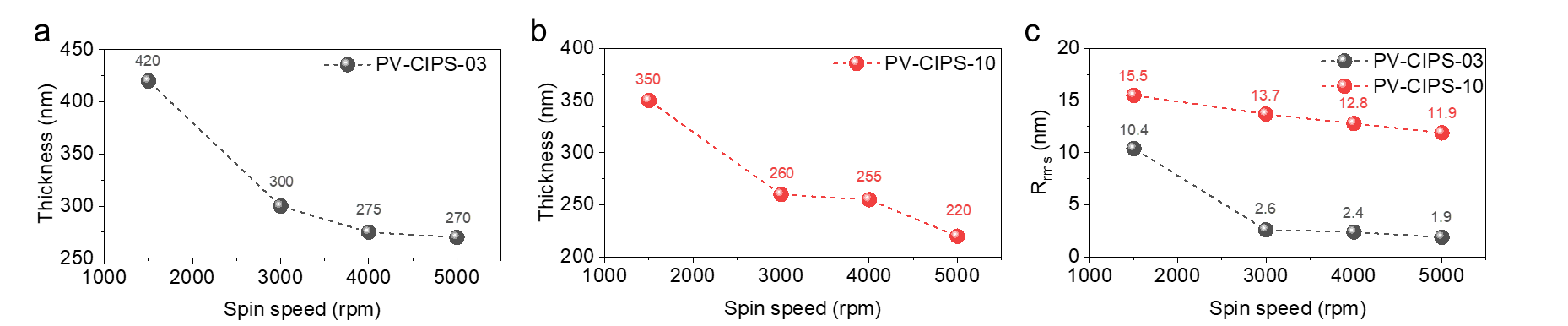
Figure S9.** Film thickness of a) PV-CIPS-03 and b) PV-CIPS-10 as a function of spin speed. c) Root-mean-square surface roughness (R_RMS_) measured over a 5 μm $\times$ 5 μm area as a function of spin speed for both PV-CIPS-03 and PV-CIPS-10. PV-CIPS-03 exhibits a pronounced reduction in roughness (from 10.4 nm to 1.9 nm), indicating improved film uniformity with increasing spin speed, whereas PV-CIPS-10 shows relatively high roughness due to increased nanosheet aggregation at higher CIPS loading.


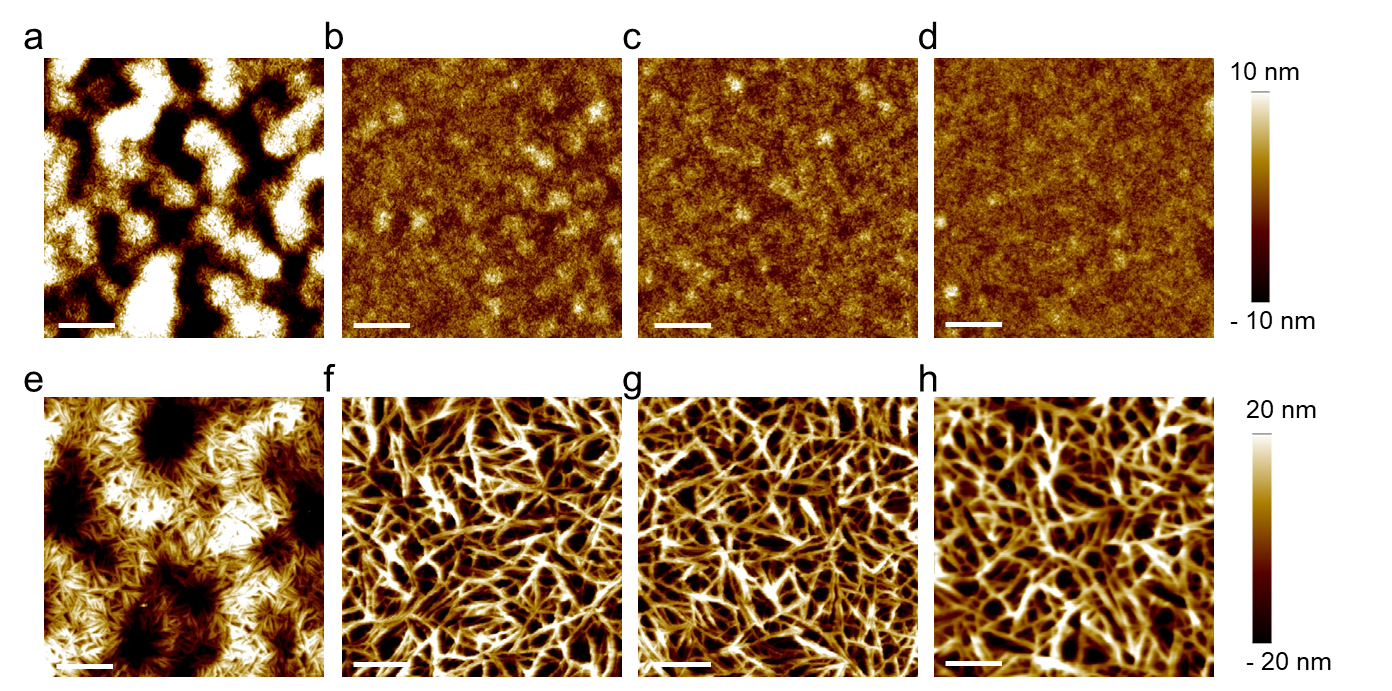
**Figure S10.** Topographical AFM images of a-d) PV-CIPS-03 and e-h) PV-CIPS-10 as a function of spin speed. a,e) 1500 rpm, b,f) 3000 rpm, c,g) 4000 rpm, and d,h) 5000 rpm. All scale bars represent 1 μm.


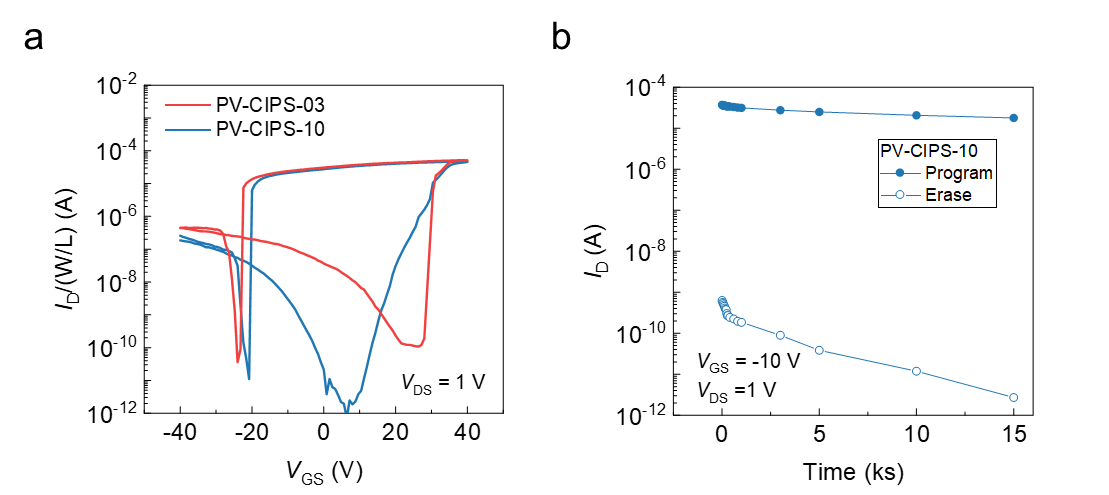


**Figure S11.** a) Transfer characteristics of PV-CIPS-03 and PV-CIPS-10 devices. b) Retention characteristics of the PV-CIPS-10 device in the Program and Erase states. Despite applying a negative gate bias of *V*_GS_ = -10 V, the Erase-state drain current (*I*_D_) decreases by nearly two orders of magnitude, reaching only a few tens of pA after 10 ks, indicating poor retention stability. In contrast, the MoS_2_/PV-CIPS-03 device maintains hole-current levels in the nA range under the erase state, as discussed in the main text (Figure 3e).


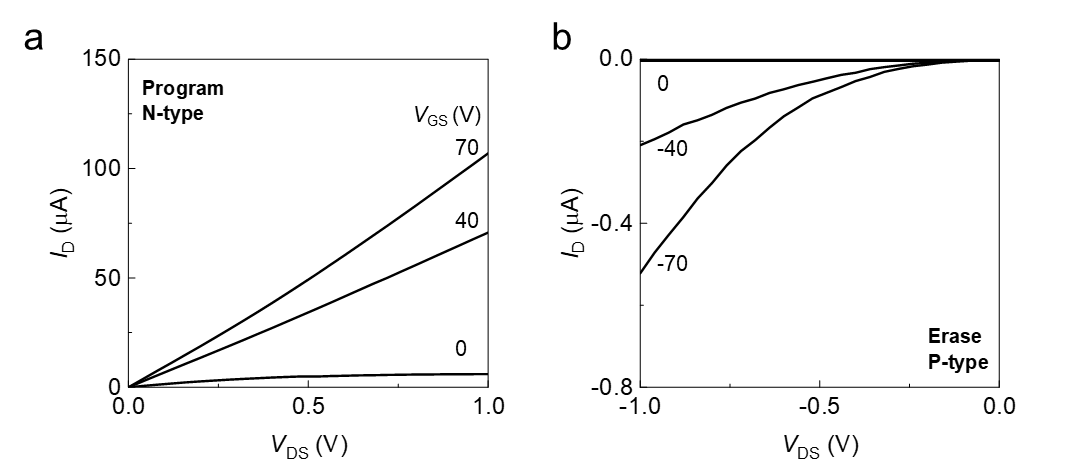


**Figure S12.** Output characteristics of the MoS_2_/PV-CIPS-03 device under a) the Program (n-type) state and b) the Erase (p-type) state. The n-type program condition exhibits linear, ohmic-like *I*_D_-*V*_DS_ behavior across *V*_GS_=0, 40, and 70 V, indicating efficient electron injection. In contrast, the p-type erase condition displays nonlinear characteristics consistent with Schottky-limited hole transport, confirming asymmetric contact behavior between the two operational states.


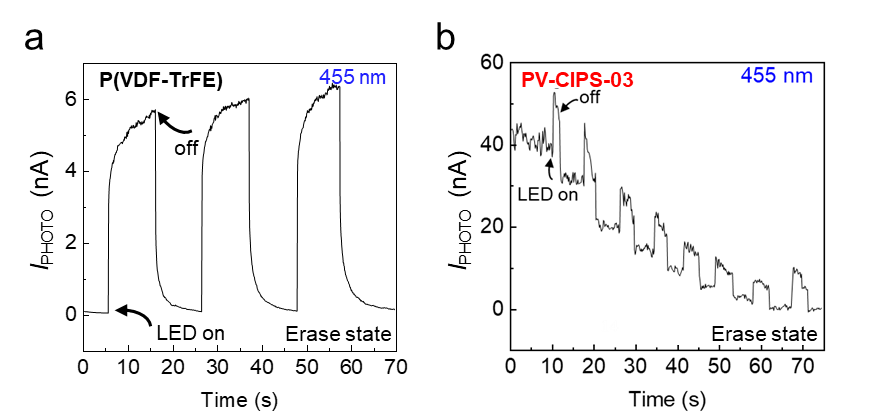


**Figure S13.** Photocurrent response of the MoS_2_/P(VDF-TrFE) control device under 455 nm illumination in the erase state. The device exhibits a stable and repeatable photoresponse without any decay in *I*_PHOTO_, indicating a conventional photoconductive behavior dominated by photo-excited electrons in MoS_2_. This stable photocurrent contrasts sharply with the gradual decay observed in the MoS_2_/PV-CIPS-03 device (Figure 4f), confirming that the decay originates from light-induced depolarization of the CIPS nanosheets rather than from the MoS_2_ channel or the P(VDF-TrFE) matrix.
